# What do countries need to do to implement effective ‘find, test, trace, isolate and support’ systems?

**DOI:** 10.1177/0141076820939395

**Published:** 2020-07-14

**Authors:** Selina Rajan, Jonathan D. Cylus, Martin Mckee

**Affiliations:** 1 Department of Health Services Research and Policy, The London School of Hygiene and Tropical Medicine, London, WC1H 9SH, UK; 2 European Observatory on Health Systems and Policies, The London School of Economics, London, WC2A 2AE, UK

Any country thinking of easing COVID-19 lockdowns must be confident that they have a robust system in place to find, test, trace, isolate and support new cases. This is essential if they are to minimise the risks of a second wave going out of control. The theory is simple. Anyone with symptoms is tested and, if positive, their contacts are traced and advised or instructed to isolate. The reality is somewhat different. It requires a complex system with many interlinking components, demanding rapid and effective communication between different organisations, some of which are newly created, while others may be combining their day-to-day work with a major expansion in capacity. Even the best resourced public health system would struggle given the scale of the pandemic. For many, especially those whose capacity has been diminished as a consequence of sustained underinvestment, the challenges are enormous. To help those who are facing these challenges, we have examined what countries across Europe are doing, seeking where possible lessons that can be learned from their experiences.

This analysis uses information gathered from the COVID Health System Response Monitor, created by the European Observatory on Health Systems and Policies,^1^ the World Health Organisation European Regional Office and the European Commission. A network of national correspondents from 50 countries has prepared a series of structured reports on national responses to the pandemic, regularly updating them as events develop.

Conceptually, we can consider a find, test, trace, isolate and support programme as a complex adaptive system, with the individual being tested passing along a non-linear route involving multiple paths, each with feedback loops and with their speed and direction influenced by a multiplicity of factors, many outside their control. Practically, however, if we are to help the busy policymaker, we must simplify this considerably, something that we have done by portraying the main elements of the system as a Snakes and Ladders boardgame (Figure 1). Snakes and Ladders is remarkably well suited to this exercise. To be successful (i.e. to win the game) countries must ensure that those with COVID-19 progress as quickly as possible from the start to the finish. If this does not happen, new cases will appear, and another lockdown will be needed. They can do this most effectively by putting in place measures that enhance their ability to find, test, trace, isolate and support (i.e. landing on ladders) and by avoiding setbacks that occur due to insufficient capacity in the health system and beyond (i.e. avoiding snakes). We now run through the boardgame, pointing out many of the steps that policymakers should be mindful of, highlighting approaches that countries are currently taking to implement a find, test, trace, isolate and support system and thereby ‘win the game’. Before doing so, however, it is important to note an important difference from the real game, in which players land on squares at the throw of a dice. In this case, countries that went into the pandemic with strong public health systems and systems of governance are more likely to land on ladders because the capacity is already in place. The role of the public is also central and unless people are made aware of the symptoms of COVID-19 and the importance of getting tested, it will be difficult to ‘find’ cases in the first place.

**Figure 1.**
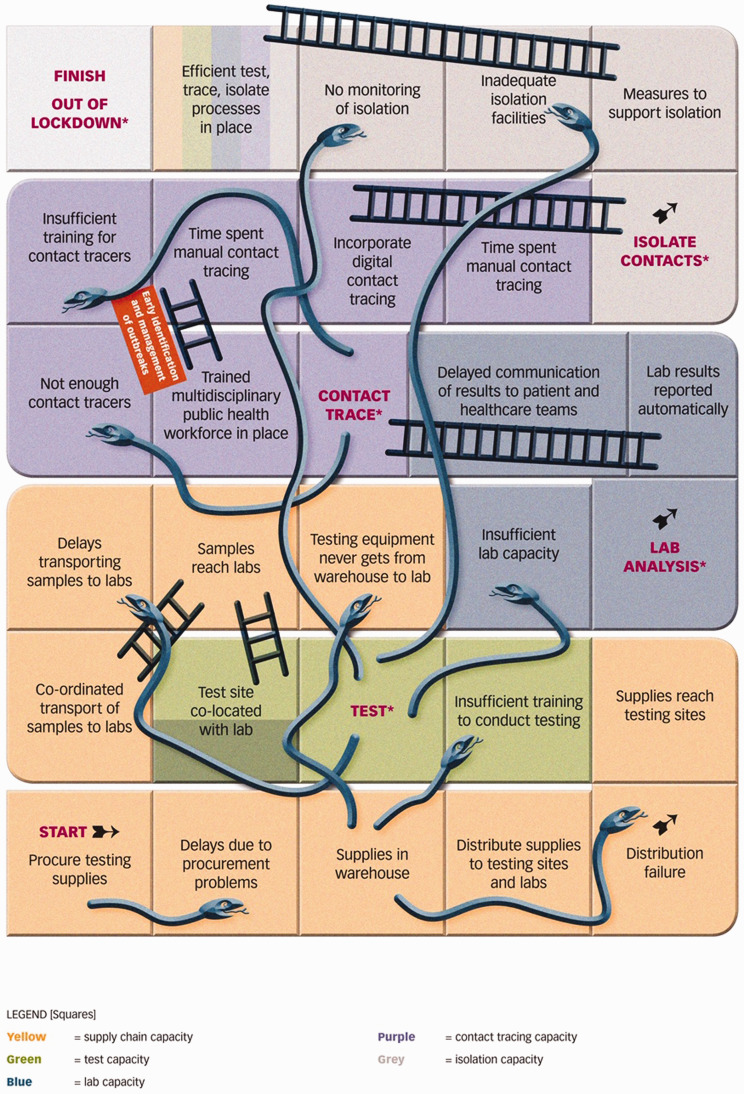
The Snakes and Ladders boardgame of find, test, trace, isolate and support.

## Producing and procuring enough testing materials

The game starts with procurement, with a focus on molecular testing supplies for nose and throat reverse transcription polymerase chain reaction swabs, the gold standard test recommended^2^ by the World Health Organization to identify COVID-19 cases. Testing requires reliable supplies of a range of materials, including swabs, transport media, reagents, primers, assays and polymerase chain reaction machines. Many of these are also used to test for other infections but, during a pandemic, countries face supply constraints, a ‘snake’ that inhibits find, test, trace, isolate and support before it has a chance to get started.

Equipped with the genetic sequence from China, Germany and the UK managed to manufacture some of the earliest COVID-19 tests outside Asia and Germany quickly purchased millions of them. Germany also published a blueprint that the World Health Organization could share with other countries to support their use of the newly developed test. However, large-scale testing is only possible if laboratories have all of the items required, from glassware to polymerase chain reaction machines. This requires very well-functioning procurement and distribution systems, something that many countries have struggled to achieve, and even Germany, widely praised for its ability to scale up testing capacity rapidly, has experienced periods when demand has exceeded supply. Countries that do not manufacture these items themselves have struggled to obtain them in a global market where they are competing against others with greater purchasing power. Some, such as Norway, have developed and manufactured their own tests^3^ to minimise dependence on those produced elsewhere. Rather like printers, where cartridges are specific to particular brands, polymerase chain reaction machines are often licensed for use with specific reagents, with global stocks of many of them rapidly depleted. In response, some countries, including Belgium, the UK and Canada eased regulations to enable more flexible use of reagents, drawing on South Korea’s earlier response to Middle East respiratory syndrome (MERS-CoV).

Once procured and warehoused, supplies need to be distributed to testing sites and laboratories. Failure to do so effectively creates a snake because testing sites cannot administer tests without the right supplies. Countries offering home testing faced logistic challenges, especially as postal services were often weakened because of staff shortages and working with social distancing. Some countries also faced particular challenges in getting tests to certain high risk settings, such as care homes, as in the UK.^4^ A failure to distribute test kits to where they are most needed will enable new cases to remain undetected, allowing transmission to continue.

## Developing sufficient skills and facilities to meet testing needs

While few countries were conducting tests outside of hospitals early in the pandemic, most now do so, for example by building drive-through or mobile testing units, while some, such as Austria, the UK and Estonia, have also started home testing. Some governments have outsourced some components of this work to private companies, for example in Finland, Estonia and the UK, although with varying degrees of success.

Although these measures can increase the volume of testing and minimise the risk of cross-contamination in hospitals, they also present enormous logistical challenges as testing supplies must be distributed to a large number of testing sites, while testing on a large scale depends on recruitment of staff who are unlikely to have experience in taking samples. Taking a nasopharyngeal swab does require some degree of training about how and (critically) when to test to reduce the risk of false-negative results. Without proper training, tests will be wasted and need to be repeated, which in turn erodes limited capacity (another snake).

After taking a swab, samples should reach the laboratory rapidly. Otherwise they may have to be discarded and repeated. Thus, it is important to ensure that there is a well-coordinated system to ensure transport of samples from test sites to laboratories. Ideally, testing sites and laboratories would be co-located, as in hospitals and in some South Korean drive-through testing sites. This is a ladder, although one that is rare in community testing sites in Europe. The ultimate goal is to develop a test that does not require a laboratory, using a point of care test that can produce immediate results, but those that have been developed so far have not performed sufficiently well to depend on. Estonia offers an innovative approach, using drones to deliver samples directly to laboratories. In the UK, approximately half of all testing takes place in just three commercial mega-laboratories, creating transport bottlenecks and reports of discarded samples.

## Strengthening laboratory capacity to rapidly analyse samples and immediately report the results

The ability to scale up testing will be easier in countries that have had sustained investment in health infrastructure, including laboratory equipment, technicians, logistics systems and information technology. Germany^5^ entered the pandemic with a strong diagnostics and chemicals industry, which allowed it to implement large scale testing rapidly.^6^ In contrast, the UK did not. Thus, a lack of sufficient laboratory capacity is another snake that will create severe delays in processing tests, possibly requiring substantial re-testing which exacerbates an already difficult situation.

Where laboratory capacity is insufficient, three types of response can be seen. One involves expanding existing medical laboratories or repurposing others, such as those involved in veterinary surveillance in universities, as in Croatia, Cyprus, France, Estonia, Germany, Lithuania and Norway, among others. Thus, Germany^5^ rapidly commissioned testing in 300 local laboratories and Sweden also used existing laboratories in all but two of its 21 regions. A second involves creation of a few centralised mega-laboratories. In contrast, in the UK, outsourcing companies, many with little or no experience of running laboratories, were contracted to construct a few mega-laboratories, creating a highly centralised system. A third approach, seen in Ireland and Finland, involved samples being sent abroad for testing, although several thousand sent from the UK to the USA subsequently had to be repeated. Other measures that also contribute include accelerated training of laboratory technicians, as in Israel, or use of robots, as in Sweden.

While there is widespread agreement that tests should be conducted within a country, where possible, debate continues as to the other approaches. Countries adopting the first one do appear to have been successful in administering and reporting large numbers of tests, whereas the situation in the UK is much less clear, with the chair of the National Statistics Authority describing the resulting statistics as essentially meaningless.

Once samples are processed, automated reporting can create a ladder, helping to deliver results quickly to cases and contact tracers who will be able to initiate tracing sooner. There are numerous examples of countries where this is working, including Belgium, Estonia, Iceland, Turkey and particularly Lithuania, where results are currently being delivered within 6–8 h. Rapid initiation of contact tracing will reduce the risk of further transmission. It also increases the likelihood that suspected cases will agree to isolate while they wait for their results. Without an automated system, results have to be telephoned individually to cases, which is resource intensive and can delay notification and isolation.

Self-evidently, there must be a system to monitor test performance to ensure false positives and negatives are minimised. This may create logistic challenges where new or repurposed laboratories have come on stream, although there are examples, such as those in Italy and Ireland, that can offer lessons.

## Building a large, well-trained workforce to conduct contact tracing (even in countries using digital technologies)

Despite renewed attention, contact tracing is a core component of public health departments, which have long experience in preventing transmission of other communicable diseases such as tuberculosis, hepatitis and sexually transmitted infections. Contact tracing requires a well-resourced existing public health infrastructure, with a trained workforce that is well connected with local services. Such a system will enable clusters and complex outbreaks to be detected early. This is an important ladder that will help to strengthen the find, test, trace, isolate and support process and is crucial for any containment or mitigation strategy. Various strategies have been used to trace contacts, outlined elsewhere^7^ but each case must be interviewed to ensure that they isolate, identify and risk assess their contacts, providing sufficient information to locate and engage with them. An inadequate number of contact tracers creates a snake as manual contact tracing is time consuming, demanding a large workforce. Any delays will lead to increased transmission.

To avoid this snake, several countries have recruited paid contact tracers to work in call centres, including France (>5000), the UK (18,000) and Germany (up to five contact tracers per 20,000 inhabitants), although a recent survey in Germany showed that only 24% of departments were able to meet this target in mid-May and it is unclear what proportion will be experienced contact tracers. There are various ways to boost the contact tracing workforce. They include inviting experienced environmental health officers, sexual health specialists and retired doctors and nurses, as the UK has done (although uptake is unknown). Others have recruited military personnel (as in Germany and the UK) and medical students (as in Finland), or recruited volunteers (as in Cyprus). However, in all cases, there can be challenges in ensuring that they are all adequately trained.

There has been considerable attention on digital technology, specifically apps as a potential ladder, given their potential to identify and notify contacts quickly. Countries where they have been implemented include Austria, Bulgaria, Canada (Alberta), Finland, Georgia, Iceland and Italy, while Germany has announced their app will be launched in mid-June. The UK, which adopted its own centralised system, unlike most other countries that used existing capabilities of mobile phones, has undertaken a pilot but the results are unknown. However, while apps may deliver speed, coverage and compliance are not guaranteed, meaning that considerable time is still required to manually trace all contacts. Recognising that digital solutions do not offer a panacea, Belgium and France have opted for manual contact tracing initially and Malta continues to debate its use, along with Finland, which is piloting robotic callers. To support the required increase in capacity, the German Ministry of Health has committed €50 million to support necessary upgrades in hardware and software. In many countries (including Austria, Belgium, Croatia, Estonia, France, Greece and Ukraine) primary care services are integrated into the test, trace, isolate process and can monitor and support cases more effectively.

## Supporting people in isolation (unless you want to start the game again)

Isolation is arguably the most important part of the ‘test, trace, isolate’ process according to recent evidence in pre-print.^8^ A team of community volunteer contact tracers in the UK published data^9^ from a pilot in which it took approximately 80 min to manage each case, with many contacts were unwilling to isolate. Measures to support isolation are therefore an important ladder and in Denmark and Finland, people who cannot isolate will be accommodated elsewhere (albeit for a fee in Finland). The same approach has also been used successfully to prevent outbreaks in care homes in South Korea. Without facilities to support vulnerable individuals to isolate, and especially to minimise any loss of income, it is likely that transmission will rise, another snake that could set back the entire process. Enforcing isolation is also critical^7^ and many countries impose fines. Some countries, including Hungary, Iceland, Italy, Lithuania, Norway and Ukraine^7^ use geolocation data to monitor the movements of cases, but such efforts still require a dedicated workforce to enforce it. This requires resources and connections to local service providers who know the local populations. Some groups^10^ have suggested that community health workers could be trained for this purpose.

## Successful ‘test, trace, isolate’ depends on having adequate capacity in many areas of the public health system

The resources required to successfully find, test, trace, isolate and support cannot be underestimated. Each step requires complex management and logistics and a well-resourced public health infrastructure and workforce. Setbacks can be encountered at any stage, but many can be anticipated. Many countries have developed innovative measures that can boost capacity rapidly. However, it is important to focus on the outcome of find, test, trace, isolate and support rather than the amount of activity. Increasing the number of tests, especially if the numbers reported do not include those actually reported on, as in the UK, will have limited value without a well-resourced system to trace and isolate cases. In addition to scale, speed is essential. Delays at any stage will allow more cases to remain under the radar, silently spreading the infection to others. Ultimately, the success of find, test, trace, isolate and support is to get countries out of lockdown. This will depend critically on their ability to be coordinated, flexible and prepared.

## Declarations

### Guarantor

SR.

### Provenance

Not commissioned; editorial review
